# Modeling of polyethylene, poly(l-lactide), and CNT composites: a dissipative particle dynamics study

**DOI:** 10.1186/1556-276X-6-433

**Published:** 2011-06-17

**Authors:** Yao-Chun Wang, Shin-Pon Ju, Tien Jung Huang, Hung-Hsiang Wang

**Affiliations:** 1 Department of Mechanical and Electro-Mechanical Engineering, Center for Nanoscience and Nanotechnology, National Sun Yat-sen University, Kaohsiung, Taiwan 804; 2 Material & Chemical Research Laboratories, Industrial Technology Research Institute, 195, Sec. 4, Chung Hsing Rd., Chutung, Hsinchu, Taiwan 31040

## Abstract

Dissipative particle dynamics (DPD), a mesoscopic simulation approach, is used to investigate the effect of volume fraction of polyethylene (PE) and poly(l-lactide) (PLLA) on the structural property of the immiscible PE/PLLA/carbon nanotube in a system. In this work, the interaction parameter in DPD simulation, related to the Flory-Huggins interaction parameter *χ*, is estimated by the calculation of mixing energy for each pair of components in molecular dynamics simulation. Volume fraction and mixing methods clearly affect the equilibrated structure. Even if the volume fraction is different, micro-structures are similar when the equilibrated structures are different. Unlike the blend system, where no relationship exists between the micro-structure and the equilibrated structure, in the di-block copolymer system, the micro-structure and equilibrated structure have specific relationships.

## Introduction

Polymer/nanomaterial composites have attracted a lot of attention because the polymer properties are significantly improved. For example, a polymer mixed with a nanolayer has higher thermal stability [1]. When the polymer is mixed with single wall carbon nanotubes (SWCNTs), the mechanical strength is substantially increased [2]. There are many nanomaterials which can be mixed with polymers, such as nanotubes (1D), clusters (0D), and nanolayers (2D). Among these nanomaterials, carbon nanotubes (CNTs) of 1D nanostructure are the most well-known material and are very promising due to their outstanding characteristics, such as high stiffness, high Young's modulus, and electronic properties. Because of this, CNTs have been proposed for several applications, such as in sensors [3,4], gas storage [5], polymer/nanotube composite materials [6-8], and as surfactants [9].

In particular, intensive efforts have been directed toward synthesizing, characterizing, and understanding polymer/CNT composites. Recent investigation has revealed many novel properties of polymer/CNT systems. Polyimide/CNT composites can reduce the softening effect of temperature, and the Young's modulus of polyimide/CNT composites in the axial direction increases 57 times over when the weight fraction of the CNTs is 16% [10]. In addition, the CNTs can reinforce the epoxy cryogenic mechanical properties at 77 K because of strong CNT/epoxy interfacial bonding. The cryogenic tensile strength, Young's modulus, and failure strain of epoxy/CNT composites are enhanced by adding 2 wt.% CNTs [11]. Because of improvements such as those above, investigations of polymer/nanotube composites are an extremely popular subject.

As a representative polymer material, polyethylene (PE) is widely used and comprises 20% of the plastic production in the world due to its numerous excellent properties, such as chemical resistance, good impact resistance, and high durability [12]. Another material, poly(l-lactide) (PLLA) is used primarily in biomedical applications such as drug delivery systems [13,14], medical sutures [15], and orthopedic materials because of its high tensile strength and higher end-use temperature. Furthermore, this material is biodegradable, thereby reducing pollution. To further improve the properties of these two materials, CNT-based nanomaterial composites are an effective strategy, leading to numerous studies by many researchers. In experiment, Zhang et al. obtained CNT/high-density polyethylene (HDPE) and CNT/ultra-high-molecular-weight polyethylene (UHMWPE) composites which alter mechanical properties by controlling PE crystallization. Compared with the mechanical properties of CNT fibers, the tensile strength and Young's moduli of CNT/HDPE and CNT/UHMWPE composites show an increase [16]. Daisuke et al. studied the effects of preparation conditions of a SWCNT/PLLA composite. They found that the SWCNT/PLLA composite has the highest dispersion in the 5 wt.% PLLA solution in chloroform. The SWCNT/PLLA composite has higher storage modulus, 3.3 times that of pure PLLA [17]. Zhang et al. found that the hydrophobic functional group (C-CH_3_) can increase the interaction between PLLA and multi-walled CNTs (MWCNTs). When the MWCNT loading is 14 wt.%, the composite has the maximum conductivity of 0.1 s/cm [18]. On the theoretical side, molecular simulations have been used to study polymer blends, di-block copolymers, and polymer/CNT composites properties [19]. Mokashi et al. used molecular dynamics (MD) to investigate the length effect on PE/CNT composites. They found that the Young's modulus and tensile strength of PE/CNT composites comprising short CNTs become smaller than that of pure PE materials [20]. Yang et al. demonstrated the adsorption structure of PE with different lengths on the CNT surface by using MD. When the length of the PE chain increases, the orientation of PE molecules adsorbed on the CNT prefers to arrange parallel to the CNT axis [21].

Although MD simulation is a widely used method, because it is limited in its time and length scales in simulation and cannot effectively prevent a configuration becoming trapped at a local minimum energy, it is difficult to observe the phase transformation process of a composite system. Dissipative dynamics particle (DPD) is an effective method to predict the structure on the mesoscopic scale. The soft forces which allow a considerable increase of time step (5 × 10-12 s) are applied in the DPD simulation [22]. In addition, DPD simulation can preserve hydrodynamics behaviors [23]. These reasons allow the system to reach the equilibrium state simply. Therefore, we chose the DPD method to predict a realistic structure.

Recently, the DPD method has been used to investigate numerous material properties in many areas, such as the phase transitions of the CNT/polymer and the polymer/polymer composites [24], the formation of micelle in the solvent [25], and the viscosity property of polymers. In our previous studies, we investigated the effect of the arrangement of the micro-structure and the effect of the volume fraction on the structural properties of the immiscible PE/PLLA/PE tri-block copolymer. The volume fraction affects the bridge and loop fraction and the equilibrium structure [26]. The different degree of functionalized-PE/CNT composites with various volume fractions (1/1, 1/4, 1/6, 1/10, 1/14, and 1/20) was also analyzed [27]. According to our previous experiments, we expect that the PE/PLLA/CNT composites could have more complex structural behaviors and demonstrate different structures. Therefore, how to accurately predict structural behavior is important. Since we have successfully predicted the structure of PE/PLLA/PE and functionalized-PE/CNT composites by DPD simulation, we have extended our previous studies to predict the structure of PE/PLLA/CNT composites. It is worth understanding how to adjust the equilibrium structure at different volume fractions and mixing methods. Consequently, in this study, the hierarchical procedures for bridging DPD and MD methods were used to study the effects of volume fractions and different mixing methods on the phase and the structural arrangement. In order to explain these effects, calculations of the gyration radius and the order parameter were used to observe the detailed arrangement of the polymer chains and the CNT, respectively, in the PE/PLLA-CNT composite system.

### Simulation method

DPD simulations were utilized to investigate the structure of PE/PLLA-CNT composite. In the DPD simulation, two important parameters, compressibility parameter and the mixing energy, were obtained from the MD simulation. Because these parameters cannot be used directly in the DPD simulation, they are transferred by coarse-grain mapping procedure after being obtained from the MD simulation. Hence, there are three detailed section parts in the simulation model. The first section is the MD simulation, the second section is the coarse-grain mapping, and the third is DPD simulation.

### Molecular dynamics simulation

**Figure 1:**
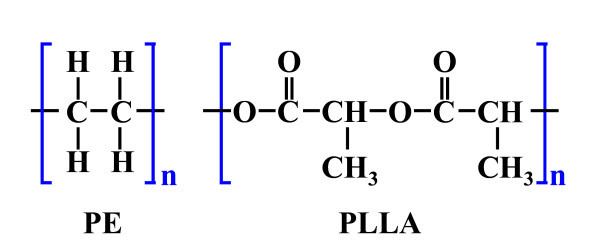
**The chemical structure of PE and PLLA**.

Molecular dynamics simulation was carried out using the Discover and Amorphous Cell module of Material Studio 4.3, developed by Accelrys Software, Inc (10188 Telesis Court, Suite 100, San Diego, CA 92121, USA). The compass potential and Andersen thermostat were used in our simulation. The time step of 1 fs was set for the time integration. Figure 1 shows the chemical structure of PLLA and PE. To calculate the compressibility, the mixing energy, and the Flory-Huggins parameter, the equilibrium structure of the CNT, PE, PLLA, CNT-PLLA, PLLA-PE, and CNT-PE composite should be obtained from MD. All processes of obtaining the interaction parameters were similar to our previous study [26]. The Flory-Huggins parameter can describe the mixing effect. The relationship between Flory-Huggins parameter and mixing energy is shown below:(1)(2)

where *R *is the gas constant and Δ*E*_mix _is the cohesive energy density which is obtained from the MD simulation as mentioned in "Molecular dynamics simulation." *ϕ*_*A *_and *ϕ*_*B *_are the volume fractions of the two components in the blended system. *V *is the volume of the simulation model and *E*coh is the cohesive energy. From the calculation above, a realistic interaction parameter between the CNT, PE, and PLLA pair in DPD can be obtained from a Flory-Huggins parameter using an atomistic simulation (MD). *V*_seg _is the volume of the polymer segment corresponding to the bead size in the MD simulation. Based on the Flory-Huggins theory, every bead has the same volume, and the polymer is assumed to be a chain that consists of several coarse-grain beads. In our MD simulation, the volume of PE with 22 PE monomers is 1,210 Å^3^, PLLA with 6 PLLA monomers is 1,150 Å^3^, and that of (5,5)CNT with 14 units is 1,243 Å^3^. Therefore, the volume of each bead is roughly set at 1,200 Å^3^, which is close to the volume of CNT and that of PE and PLLA.

The dimensionless compressibility method was obtained from the slope of the line from Ref. [16]. Hence, to obtain the corresponding number density at different target pressures, the PLLA equilibrium structure derived from the NVT MD simulation is used as a base to continue the NPT simulation at different target pressures at 300 K. A 200-ps NPT MD simulation is performed to equilibrate the structure of PLLA polymer system and then to obtain the corresponding number density.

### Coarse-grain mapping

In the DPD simulation, the total force acting on a DPD bead *i *is expressed as a summation over all the other beads, *j*, of the conservative force, a dissipative force, a random force, and a spring force. The conservative force is a soft repulsive force, where the interaction strength of this repulsive force is determined by the repulsive interaction parameter (*a*_*ij*_). When bead *i *and *j *are the same substance, the repulsive interaction parameter is obtained from the compressibility parameter. In "Molecular dynamics simulation," MD is used to calculate the compressibility parameter from the PLLA polymer system, which we then match to the DPD system's dimensionless compressibility [23]:(3)

where *ρ *is the number density, *N*_m _is the coarse-grained parameter, *k*_B _is the Boltzmann constant, and *T *is the system temperature. The meaning of *N*_m _is the number of molecules in one DPD bead. In this study, the number of PE molecules in one bead is 1. Then, the repulsive parameter (*a*_*ii*_) of the same kind of polymer can be determined from the relationship between *a*_*ii *_and the dimensionless compressibility parameter, which is found in a reference from Groot and Warren:(4)

It should be noted that Equation 3 only establishes when the number density (*ρ*) is larger than 2. In order to simulate more efficiently, we chose the minimal value of 3. Groot and Warren's study shows that they can insert the mixing effect as Δ*a *into the repulsive interaction parameter *a*_*ij *_for different kind of beads by the Flory-Huggins parameter *χ *which is obtained from the MD simulation. For the case in which the reduced density *ρ *is 3, this relationship is as follows:(5)(6)

From Ref. [24], the repulsive interaction parameter in the DPD simulation can be used to obtain the surface tension. However, because this value unmodified is not accurate since the surface tension of experimental data is a constant, they assumed that a range of Δ*a *has a linear variation between 15 and 115, with a χ value of 0.3 at Δ*a *Δ*a *= 15 and a value of 0.2 at Δ*a *= 15. After modifying Δ*a*, the surface tension is a constant and is close to the experimental data.

### Dissipative particle dynamics simulation method

In the present research, the DPD simulation method was adopted to investigate the effect of volume fraction of a PE/PLLA/CNT composite on the structural property. Equations 7 and 8 describe the condition that the DPD simulation follows Newton's equation of motion:(7)(8)

However, in a DPD simulation, all of the beads in the system are of the same volume regardless of the number of and kinds of different molecules comprising the beads. This assumption is required because the system must conform to the Flory-Huggins *χ*-parameter theory [23]. For simplicity, the masses of all particles in the system are normalized to 1. Equation 9 represents the fact that the total force consists of four forces. The interaction force on bead *i *is given by the sum of a conservative force , a dissipative force , a random force , and a spring force .(9)

where conservative force represents a purely repulsive force, dissipative force represents the friction between DPD beads that reduces velocity differences between the particles, random force works to conserve the system temperature, and the spring force is used to bind the intra-polymer beads. The second and third forces are responsible for the conservation of total momentum in the system. All of the forces act within a sphere of cutoff radius *r*_*C*_, which also defines the system's length scale. The conservative force with a linear approximation is given by:(10)

where *r*_*ij *_is the distance between bead *i *and bead *j*, and *a*_*ij *_is the repulsive interaction parameter describing the interaction strength between beads. When *i *material is the same as *j *material, the repulsive interaction parameter is obtained from the dimensionless compressibility parameter (Equations 3 and 4). Moreover, when *i *and *j *materials are different, the repulsive interaction parameter is obtained from Equation 6 and Δ*a *is obtained from the Flory-Huggins *χ*-parameter theory from MD simulation.

In our DPD simulation, the cell volume is 20 × 20 × 20 and the number density of the system is 3 (*ρ *= 3). The system contains 24,000 beads. It consists of 250 chains, every chain consisting of 12 beads. The chain length is fixed at 12 beads at every volume fraction (including 1/1, 1/4, 1/6, 1/14, and 1/20). We can adjust the bead ratio to reach the different volume fractions. In order to describe the structure of the CNTs, the potential of the bond extension and angle were performed for the CNT and shown as follows:(11)(12)

where *C*_*b *_and *k*_*a *_are force constants representing the bond stretch and bond bending, respectively, and *θ*_*a*_, *r*_*b*_, *θ*_*a*_^0^, and *r*_*b*_^0 ^are the bending angle, the length, the equilibrium angle of the bending angle, and the equilibrium length of the bond.

## Result and discussion

Before performing the DPD simulation, the repulsive interaction parameters should be obtained first and are listed in Tables 1, 2, and 3 for 10/10/1, 6/14/1, and 2/18/1 volume fractions, respectively. In the DPD simulation, all the repulsive interaction parameters between the same materials are 38.403. When the repulsive interaction parameter between different materials is larger than that between the same material, it means that these two materials have stronger repulsive interaction.

**Table 1:** The repulsive interaction parameter at 10/10/1 volume fraction

10:10	CNT	PE	PLLA
CNT	38.403	38.51	139.33
PE	38.51	38.403	106.474
PLLA	139.33	106.474	38.403

**Table 2:** The repulsive interaction parameter at 6/14/1 volume fraction

6:14	CNT	PE	PLLA
CNT	38.403	45.871	188.134
PE	45.871	38.403	131.9
PLLA	188.134	131.9	38.403

**Table 3:** The repulsive interaction parameter at 2/18/1 volume fraction

2:18	CNT	PE	PLLA
CNT	38.403	82.35	217.068
PE	82.35	38.403	349.36
PLLA	217.068	349.36	38.403

From Table 1, 2, and 3, we can observe that the repulsive interaction parameter between PE polymers and CNTs decreases with an increase in PE polymer volume fraction. It indicates that the CNTs are easily dispersed into the polymer matrix at a lower CNT fraction. At a lower polymer fraction (a higher CNT fraction), the much higher repulsive parameters between PE and CNT beads lead to the aggregation of CNTs surrounded by the polymer matrix. The characteristic of repulsive parameters at different fractions corresponds to the related experimental observation. Chen et al. demonstrated that CNTs with smaller weight fraction in the polymer matrix will be easily dispersed [11]. In addition, we found that the repulsive interaction parameter between PLLA and PE polymers increases from 6/14/1 to 2/18/1 volume fractions. The reason for this is that the calculation of cohesive energy density includes the weight function for a pure component, which is shown in Equation 2.

**Figure 2:**
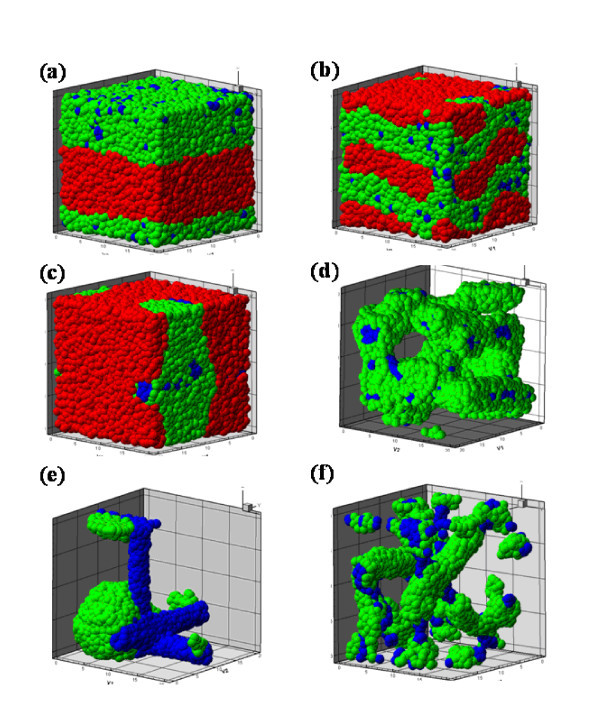
**The equilibrated structure at (a-b) 10/10/1, (c-d) 6/14/1, and (e-f) 2/18/1 fractions**.

After the DPD simulation was performed, all equilibrated structures were obtained at different volume fractions with blend and di-block copolymer methods, which can be seen in Table 4. All equilibrated structures of different volume fractions with these two methods are shown in Figure 2a-f. The red, green, and blue beads represent the PLLA, PE polymers, and CNTs, respectively. Figure 1a shows the lamellae structures, which are found in the 10/10/1 volume fraction in the blend method. In many DPD studies, most of the equilibrated structure is lamellae structure. However, for the corresponding di-block copolymer system, the polymer beads will form the perforated lamellae structure in the polymer/CNTs bead matrix, as shown in Figure 2b. In Figure 2a, the PE polymers and CNTs aggregated and formed one layer, and PLLA polymers formed another layer by themselves because of the relationship of repulsive interaction parameters. The CNTs did not aggregate and form the cylindrical shape because of the similar repulsive interaction parameter between the CNT and PE polymers. In addition, the value of that between PE polymer and CNT is obviously smaller than both that between PLLA polymers and CNTs and between PLLA and PE polymers at 10/10/1 volume fraction. This means that the PLLA polymer has a very strong repulsive interaction to PE and CNTs. Therefore, PLLA polymers form one layer by themselves, excluding other materials. Because CNTs with similar repulsive interaction parameters were not forced to connect to PE or PLLA polymers, CNTs also disperse inside the PE polymer matrix. From Figure 2b, we found that the layer in Figure 2b is thinner than that in Figure 2a. In the di-block copolymer method, one PE polymer chain was forced to connect to a PLLA polymer chain, and the movement of these two polymers is restrained in the polymer/CNTs matrix. For example, the PE polymer only can adsorb on the PE side of other di-block copolymer chain and arrange parallel to form the perforated lamellae structure. However, in the blend method, every material can aggregate together easily because they do not have any movement limitations. Therefore, the thickness of the layer in the blend method was larger than that of the di-block copolymer method.

**Table 4:** The equilibrated structure at three volume fractions with blend and di-block copolymer methods

PE/PLLA/CNT	2/18/1		6/14/1		10/10/1	
	Blend	Di-block	Blend	Di-block	Blend	Di-block
Equilibrated structure	Cluster	Tube-like	Perforated lamellae	Tube-like	Lamellae	Perforated lamellae

**Figure 3:**
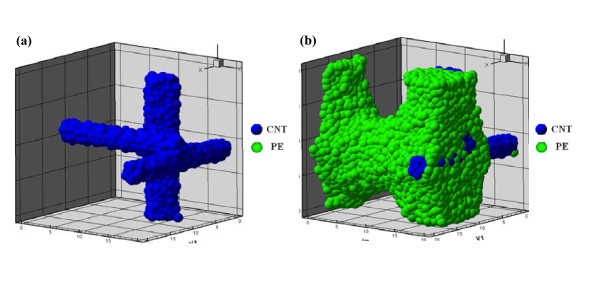
**The equilibrated structure at 6/14/1 volume fraction with blend method**.

Figure 2c,d shows the equilibrated structures, which are perforated lamellae and tube-like structures at 6/14/1 volume for blend and di-block copolymer methods, respectively. Figure 3a shows the CNT structure which forms three cylindrical structures. Compared to Figure 2a, the CNTs do not disperse at this volume fraction. The reason for this is that the repulsive interaction parameter between CNTs and PE polymers is larger than that between the same materials. As can be seen from Table 3, the repulsive interaction parameter between PLLA polymer and CNTs is the largest, and that between PLLA and PE polymer is just smaller than that between PLLA and CNTs. Therefore, there are two possible structural types for the CNTs in the polymer/CNT matrix. First, they form the cylindrical structure and are covered by PLLA polymers. Second, they are surrounded by PE polymers, and these PE polymers are surrounded by PLLA polymers. Figures 3b and 2d show the two structural types in the polymer/CNTs matrix. In Figure 2d, almost all of the CNTs are surrounded by PE polymers. This is due to the restrained movement and the relationship of repulsive interaction parameters. It is impossible for CNTs to exist in the middle of PE and PLLA polymers because of the connection between PE and PLLA polymers. In addition, the repulsive interaction parameter between PE and CNT is significantly smaller than that between PLLA and CNT. Therefore, CNTs can only be inside the PE polymers which are covered by the PLLA polymers.

Figure 2e,f illustrates the equilibrated structures at 2/18/1 volume fraction with blend and di-block copolymer methods. In the blend method, the PE polymers aggregate themselves to form the cluster because of the unrestrained structure and the lower volume fraction. Similarly, the CNTs form cylindrical structures were similar to the 6/14/1 volume fraction. In addition, there are the fewest PE polymers at the 2/18/1 volume fraction such that PE polymers do not cover all CNTs. Figure 2f shows an equilibrated structure similar to that in Figure 2d. The reason for forming the same equilibrated structure is almost the same. Because the number of PE polymers is the lowest, they cannot cover all of the CNTs. Hence, some CNTs are in contact with the PLLA polymers. In addition, the CNTs form more cylindrical structures and the PE polymer of the di-block copolymer can easily cover the CNTs.

In order to analyze the relationship between the micro-structures of PE and PLLA polymers and equilibrated structures, the square radius of gyration *Rg*^*2 *^is examined to provide information on the mass distribution of the chain in the system, which also plays a central role in interpreting light scattering and viscosity measurements. If all beads have the same mass:(13)

where *r*_*i *_denotes the coordinate of the particle, *r*_*c *_denotes the coordinate of center of mass of the polymer chain, and *n *is the bead number in a chain. Additionally, it can be represented as the tensor in different directions as follows:(14)

where *r*_*ix *_and *r*_*iy *_denote the position vector of the particle *i*, whereas *r*_*cx *_and *r*_*cy *_denote the position vector of the center of mass of polymer chain. The three eigenvalues of *G *are denoted by *Rg*_1_^2 ^(major axial, which is the largest eigenvalue) *Rg*_2_^2^, and *Rg*_3_^2^, which can be used to determine roughly the structural arrangement of a chain in the system. If the values of *Rg*_2_^2 ^and *Rg*_3_^2 ^are almost the same, it means that the micro-structure of this material is spherical structure. The summation of *Rg*_1_^2^, *Rg*_2_^2^, and *Rg*_3_^2 ^is *Rg*^2 ^which can be used to determine roughly the structural arrangement of a chain in the system. The larger *Rg*^2 ^means that the structure is extended, whereas the lower dimension phase has the more collapsed structure in the polymer chain. When the *Rg*_1_^2 ^is larger than *Rg*_2_^2 ^and *Rg*_3_^2^, the micro-structure of material is ellipsoid structure. All of the values of PE and PLLA polymers with two methods at different volume fractions are listed in Table 5. All micro-structures of PE and PLLA polymers are spherical at three volume fractions because all polymers are not restricted, and it is easy for polymers of the same material to aggregate by themselves because they have the same repulsive interaction parameter. Hence, they have the similar micro-structure at three volume fractions.

**Table 5:** The radius of gyration at three volume fractions with blend and di-block copolymer methods

PE/PLLA/CNT	2/18/1		6/14/1		10/10/1	
Blend	PE	PLLA	PE	PLLA	PE	PLLA
*Rg*_1_^2^		0.461	0.128	0.352	0.244	0.244
*Rg*_2_^2^		0.459	0.128	0.349	0.243	0.243
*Rg*_3_^2^		0.457	0.122	0.338	0.223	0.224
Di-block copolymer	PE-PLLA		PE-PLLA		PE-PLLA	
*Rg*_1_^2^	0.804		1.054		1.672	
*Rg*_2_^2^	0.713		0.859		0.6398	
*Rg*_3_^2^	0.623		0.75		0.518	
*Rg*^2^	2.14		2.663		2.829	

From the value of Table 5, all micro-structures of entirely di-block copolymers are ellipsoid in structure. The ellipsoid structures elongate with the increase of PE volume fraction. We use the ratio of *Rg*_1_^2^/*Rg*_2_^2 ^and *Rg*_1_^2^/*Rg*_3_^2 ^to compare their micro-structures. When the equilibrated structure is a perforated lamellae structure, the ratios are 0.38 and 0.32 at 10/10/1 volume fraction. When the equilibrated structure is tube-like, the ratios are about 0.8 at 2/18/1 and 6/14/1 volume fractions. This can be attributed to the different equilibrated structure at three volume fractions. The equilibrated structure at 10/10/1 volume fraction is perforated lamellae. The micro-structure of the entirely di-block copolymer is the longest and thinnest. In particular, this shape can arrange parallel to form the perforated lamellae structure. When the volume fraction of PE polymer increases, the equilibrated structure changes from perforated lamellae to the tube-like structure. If the micro-structure is thin and elongated, it is difficult for PLLA polymers to fill the spaces which are not occupied by the PE polymer and CNTs. Hence, it is easy for the shorter and wider ellipsoid structure to form the tube-like structure. Unlike the blend system, where no relationship exists between the micro-structure and the equilibrated structure, in the di-block copolymer system, the micro-structure and equilibrated structure have specific relationships such as the long and thin ellipsoid forming the perforated lamellae. It is difficult in the blend system to find the relationship between micro-structures and equilibrated structures due to the unrestricted conditions between PE and PLLA polymers.

## Conclusion

This study investigates equilibrated structure of PE/PLLA/CNT composites at different volume fractions with the blend and di-block copolymer methods. The volume fraction and mixing methods clearly affect the equilibrated structure. However, the micro-structures are only affected by the equilibrated structure in the di-block copolymer method. Even if the volume fraction is different, micro-structures are similar when the equilibrated structures are the different. In the blend method, all micro-structures at different volume fractions are spherical in structure. Possible future investigations could include the relationship between the length and micro-structure at different volume fractions.

## Competing interests

The authors declare that they have no competing interests.

## Authors' contributions

HHW carried out the molecular dynamics and dissipative particle dynamics simulations and performed the data analyse. YCW drafted the manuscript and participated in its design. TJH participated in the design of the study. SPJ participated in the design of the study and conceived of the study. All authors read and approved the final manuscript.
